# Flexural performance and microstructural correlation of natural silk fabric-reinforced epoxy composites

**DOI:** 10.1038/s41598-026-55528-3

**Published:** 2026-06-02

**Authors:** Sampath Suranjan Salins, Sawan Shetty, Deepak Doreswamy, Thamme Gowda, H. K. Sachidananda

**Affiliations:** 1 School of Engineering and IT, Manipal Academy of Higher Education, Dubai Campus, Dubai, UAE; 2 Manipal Institute of Technology, Manipal Academy of Higher Education, Manipal, 576104 India; 3 Department of Mechanical Engineering, Vidyavardhaka College of Engineering, Mysuru, Karnataka India

**Keywords:** Silk fiber-reinforced composites, Epoxy matrix, Epoxy resin chemistry, Three-point bending test, Flexural properties, Natural fiber composites, Sustainable materials chemistry, Scanning electron microscopy (SEM), Engineering, Materials science

## Abstract

This study investigates the flexural behaviour and microstructure–property relationships of natural silk fabric–reinforced epoxy composites fabricated using a cast moulding route. Three rectangular specimens (100 × 20 × 5 mm) were tested under three-point bending in accordance with ASTM D790 with a 60 mm support span. The composites were produced at moderate fibre volume fractions (approximately 20–40 vol%), and their density and void content were evaluated using mass–volume measurements and optical image analysis. The measured flexural strength ranged between 100 and 130 MPa, with results reported as mean values along with standard deviation. The composites exhibited a pseudo-ductile response characterized by an initial linear elastic region followed by progressive damage and gradual post-peak softening. Although peak stresses were comparable, clear differences in strain-to-failure and post-peak stability were observed, indicating that deformation behaviour is governed primarily by interfacial integrity and microstructural heterogeneity. Multiscale characterization using optical microscopy and scanning electron microscopy (SEM) was carried out to establish structure–property relationships. Optical observations revealed that voids and non-uniform resin impregnation influence crack initiation and propagation. SEM-based fractography identified dominant failure mechanisms including matrix cracking, interfacial debonding, fibre pull-out, and localized fibre fracture. Specimens with improved impregnation showed better interfacial continuity and crack-bridging, whereas defect-rich regions exhibited premature damage initiation and unstable crack growth. The results demonstrate that load transfer and flexural stability are governed by interfacial stress transfer and processing-induced heterogeneity. The study provides a mechanistic interpretation of flexural behaviour and offers a basis for positioning silk–epoxy composites within natural fiber reinforced systems.

## Introduction

Silk–epoxy composites represent an emerging class of bio-based structural materials that combine the mechanical performance of natural silk fibers with the stiffness and durability of thermosetting epoxy matrices. Among natural silks, *Bombyx mori* fibroin is widely recognized for its high tensile strength, toughness, and biocompatibility, making it a suitable reinforcement for polymer composites (Altman et al.^1^. When incorporated into epoxy systems, silk fibers can improve flexural strength, fracture resistance, and energy dissipation through effective stress transfer and interfacial compatibility (Chang et al.^2^. In addition, silk is renewable and biodegradable, aligning with sustainability objectives in materials engineering. Prior studies have shown that composite performance is highly sensitive to processing parameters such as fiber dispersion, alignment, degassing, and curing, which directly influence void formation and interfacial integrity (Eshkoor et al.^3^. The flexural behavior of silk–epoxy composites under three-point bending is particularly relevant for structural applications. Youssef et al.^4^ reported anisotropic flexural behavior in woven silk/epoxy laminates produced via VARTM, with a 23% improvement in specific flexural strength compared with glass/epoxy systems. However, retained moisture significantly reduced mechanical performance, emphasizing the importance of fiber conditioning. Kang et al.^5^ demonstrated that flexural strength increases with fiber volume fraction, with *Antheraea pernyi* silk achieving a threefold increase and ductile behavior at 60 vol% reinforcement. Similarly, Zuraidah et al.^6^ observed maximum flexural strength at 30 wt% fiber loading, attributed to improved interfacial bonding, although higher fiber content increased moisture absorption. These findings indicate that fiber type and reinforcement level strongly influence deformation and failure behavior.

Beyond flexural loading, silk–epoxy systems exhibit notable energy absorption characteristics. Ataollahi et al.^7^ showed that woven silk/epoxy composite tubes display geometry-dependent crushing behaviour, while Eshkoor et al.^8^ identified progressive failure mechanisms involving tearing, buckling, and delamination. Although these studies demonstrate the capacity of silk composites for energy dissipation, they primarily focus on impact or compression loading at high fibre contents. Consequently, flexural behaviour at moderate reinforcement levels, which are more relevant for processing control and defect minimization, remains insufficiently explored. Microstructural characterization has been widely used to interpret structure–property relationships. Optical microscopy reveals fibre distribution, resin-rich regions, and voids, while SEM provides insight into interfacial bonding and fracture mechanisms. Youssef et al.^4^ showed that improved resin infiltration reduces void content and enhances mechanical performance, whereas moisture adversely affects fibre wetting. Chaisomkul et al.^9^ reported that increasing fabric content improves packing but introduces resin-rich regions that promote brittle fracture. SEM studies by Kang et al.^10,11^ further demonstrated that interfacial quality governs crack deflection, fibre pull-out, and overall energy dissipation.

Recent advances in hybrid fiber–epoxy composites demonstrate a growing emphasis on multifunctional performance through strategic material integration. For instance, Huang et al.^12^ hybrid carbon fiber silk EMI composites develop a hybrid system combining carbon fiber fabric, silk fiber non-woven fabric, and carbonyl iron powder within an epoxy matrix, showing that the synergistic interaction between conductive and fibrous phases significantly enhances electromagnetic interference shielding while maintaining mechanical integrity (Huang et al.,^12^. Complementing this, Zheng et al.^13^ nano-filler silk epoxy composites examine nano-filler reinforced silk fiber-epoxy composites and report that the incorporation of nanoscale fillers improves both thermal stability and mechanical performance, with variations depending on filler type and dispersion characteristics. Extending the hybridization approach to natural–synthetic fiber systems, Behera et al.^14^ jute Kevlar SiC epoxy composites demonstrate that silicon carbide filler content plays a critical role in governing the mechanical strength, thermal resistance, and structural properties of jute/Kevlar epoxy composites, highlighting the importance of filler optimization in hybrid architectures. Collectively, these studies underscore that tailored hybridization of fibers and fillers within epoxy matrices is central to achieving balanced mechanical, thermal, and functional properties, although performance remains highly sensitive to material composition and interfacial interactions.

From a chemical perspective, silk fibroin contains amide and hydroxyl functional groups capable of interacting with epoxy matrices through secondary bonding mechanisms. While these interactions are expected to influence interfacial adhesion, their role in governing stress transfer and damage evolution under flexural loading is not fully established, particularly at moderate fibre volume fractions. A review of the literature indicates three specific gaps. First, most studies emphasize high fibre volume fractions (typically above 50–60%), whereas moderate reinforcement levels (approximately 20–40%) remain underreported despite their relevance to manufacturability and defect control. Second, prior work often reports mechanical properties without integrating them with systematic microstructural analysis across multiple length scales. Third, the relationship between processing-induced heterogeneity, interfacial integrity, and pseudo-ductile flexural response has not been explicitly established.

To address these gaps, the present study develops silk fabric–reinforced epoxy composites using controlled fabrication conditions and evaluates their flexural behavior under standardized three-point bending. By combining mechanical testing with optical microscopy and SEM-based fractography, this work establishes direct correlations between stress–strain response, microstructural features, and failure mechanisms. The study contributes by (i) linking flexural response to damage evolution across multiple scales, (ii) identifying the transition between interface-dominated and fiber-dominated failure at moderate reinforcement levels, and (iii) demonstrating that processing-induced heterogeneity, including void content and resin distribution, governs the observed pseudo-ductile behavior. These findings provide a clearer mechanistic basis for designing silk-based composites for lightweight structural applications.

## Methodology

### Composite Sample preparation

**Fig. 1:**
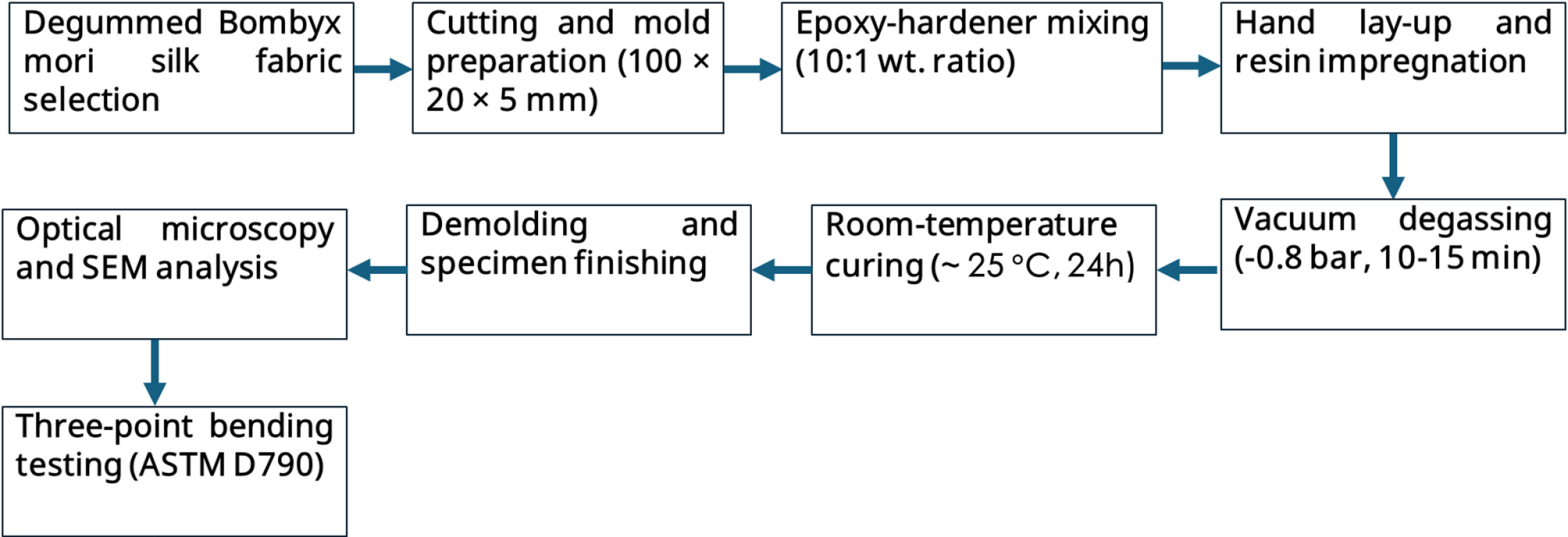
Schematic illustration of the fabrication and testing workflow for natural silk fabric–reinforced epoxy composites, including fabric preparation, resin–hardener mixing, hand lay-up, vacuum degassing, curing, specimen cutting, and three-point bending evaluation.

**Fig. 2:**
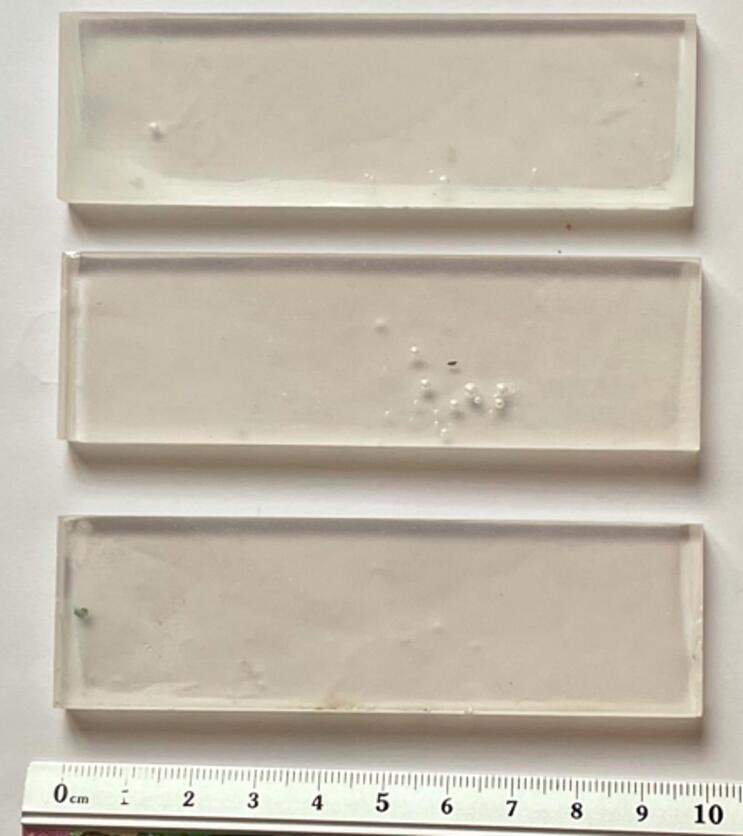
Silk fabric–reinforced epoxy composite specimens fabricated using the hand lay-up method, illustrating variations in surface finish, resin impregnation, and visible void content across samples.

Degummed *Bombyx mori* silk fabric was used as reinforcement, and a commercial bisphenol-A-based epoxy resin system with a stoichiometric amine hardener served as the matrix (Refer Fig. 1). The fabric was used without additional chemical surface treatment; however, it was conditioned by drying prior to fabrication to minimize moisture content, given the sensitivity of silk fibres to environmental humidity. The composite specimens were fabricated using a hand lay-up technique followed by vacuum-assisted degassing and room-temperature curing (Refer Fig. 2). The silk fabric was cut into rectangular plies corresponding to the mold dimensions (100 mm × 20 mm × 5 mm). A total of multiple layers of silk fabric (plain weave, typical areal density in the range of 40–60 g/m²) were used to achieve the target fibre volume fraction (20–40 vol%), and the corresponding fibre weight and volume fractions were calculated based on measured mass and known material densities. The epoxy resin and hardener were mixed in a 10:1 ratio by weight, as specified by the manufacturer, and stirred under controlled conditions to reduce air entrapment. The resin was then poured over the stacked fabric layers to ensure uniform impregnation. Curing was carried out under ambient laboratory conditions (temperature ≈ 25 °C; relative humidity estimated in the range of 50–65%), without active environmental control. The silk fabric used in this study was plain weave architecture; however, the exact areal density (GSM) was not specified by the supplier. The reinforcement level was therefore controlled and reported in terms of measured fiber weight and calculated volume fraction.

The filled mold was subjected to vacuum at approximately − 0.8 bar for 10–15 min to remove entrapped air. No external mold pressure was applied beyond vacuum conditions. The system was then cured at room temperature (approximately 25 °C) for 24 h under ambient laboratory humidity conditions. Post-curing was not performed. Thickness control was maintained using a fixed mold cavity, ensuring consistent specimen geometry across all samples. Following curing, the laminates were demoulded and cut into test specimens of 100 mm × 20 mm × 5 mm. Composite density was determined using mass-to-volume measurements, and void content was estimated through optical image analysis. Three specimens were prepared, exhibiting variations in surface quality and internal void distribution arising from processing conditions. While the sample size is limited, the results are reported as mean values with standard deviation to indicate variability. The observed differences are interpreted in conjunction with detailed multiscale microstructural characterization rather than statistical inference. From a material perspective, the epoxy curing process generates secondary hydroxyl groups, which may facilitate intermolecular interactions with polar functional groups present in silk fibroin. These interactions are considered as plausible contributors to interfacial adhesion and are discussed in relation to the observed load transfer and damage evolution under flexural loading, without implying direct chemical verification.

### Effect of fiber fraction on silk-epoxy composite properties

The fibre volume fraction is a key parameter governing the mechanical response of silk–epoxy composites, as it directly influences load transfer, interfacial stress distribution, and damage evolution. Prior studies have shown that increasing silk fibre content generally improves flexural strength, stiffness, and energy absorption due to enhanced fibre participation in load bearing. For instance, composites with relatively high silk content (60–70 vol%) exhibit improved flexural modulus and fracture resistance, attributed to increased resistance to crack propagation and more effective fibre–matrix interaction^5^. However, these improvements are not monotonic. At higher fibre fractions, limitations in resin infiltration and increased void formation can reduce interlaminar bonding and compromise structural integrity^15^. SEM observations in such systems often reveal localized interfacial debonding and fibre pull-out associated with incomplete wetting, indicating that mechanical performance is strongly dependent on processing quality in addition to fibre content. In contrast, moderate fibre volume fractions (approximately 20–40 vol%) provide a balance between reinforcement efficiency and processability. At these levels, improved resin impregnation reduces void content and enhances interfacial continuity, which can lead to more stable crack propagation and improved deformation tolerance under flexural loading. This highlights that optimal performance is governed not only by fibre fraction but also by the interaction between processing conditions, interfacial adhesion, and defect distribution.

From a material perspective, *Bombyx mori* silk fibroin fibres exhibit a Young’s modulus in the range of 5–17 GPa, depending on reeling conditions, degumming, and environmental factors, with a Poisson’s ratio typically between 0.30 and 0.35. These fibres also possess high toughness and elongation at break (up to 15–20%), which contribute to energy dissipation mechanisms such as fibre bridging and pull-out. In comparison, thermosetting epoxy resins typically exhibit a modulus in the range of 2.5–4 GPa and a Poisson’s ratio between 0.33 and 0.38, reflecting their relatively stiff but brittle nature. The combination of ductile silk fibres with a brittle epoxy matrix results in a composite system capable of improved fracture resistance and progressive damage behaviour, provided that adequate interfacial bonding and uniform microstructure are achieved.

**Table 1:** Key parameters, representative values, and governing equations used to estimate fiber volume fraction and composite modulus of silk fabric–reinforced epoxy composites based on the Rule of Mixtures^5,10,15,16^.

Parameter	Symbol	Typical Value	Units	Formula / Description
Young’s modulus of fiber	E_f_	10–17	GPa	From literature (Bombyx mori silk fiber)
Young’s modulus of matrix	E_m_	2.5–4	GPa	Typical for bisphenol-A based epoxy resin
Fiber volume fraction	V_f_	0.2–0.6	—	Input or computed from weights and densities
Matrix volume fraction	V_m_ = 1 - V_f_	Complement of V_f_	—	Automatically computed once V_f_ is known
Composite modulus	E_c_	Depends on inputs	GPa	Ec = V_f_ E_f_ + V_m_ E_m_ (Rule of Mixtures - longitudinal)
Fiber density	ρ_f_	~ 1.30	g/cm³	For degummed Bombyx mori silk
Matrix density	ρ_m_	~ 1.20	g/cm³	For cured epoxy
Fiber weight fraction	W_f_	e.g., 30	% or g	Known from material composition
Matrix weight fraction	W_m_ = 100 - W_f_	e.g., 70	% or g	Complement of W_f_
Volume fraction formula	V_f_	See formula	—	V_f_ = (W_f_/ρ_f_) / ((W_f_/ρ_f_) + (W_m_/ρ_m_))

For a silk fibre weight fraction of 30%, and assuming typical densities for *Bombyx mori* silk (1.3 g/cm³) and epoxy resin (1.2 g/cm³), the corresponding fibre volume fraction was calculated to be approximately 28.35% using a density-based conversion. Based on this value, the composite modulus was estimated using the Rule of Mixtures, taking representative fibre and matrix moduli of 12 GPa and 3 GPa, respectively. The resulting theoretical modulus of approximately 5.55 GPa indicates a moderate increase in stiffness due to fibre reinforcement; however, this value represents an idealized upper-bound estimate assuming uniform stress transfer and perfect interfacial bonding. To further interpret the mechanical behaviour of the silk–epoxy system, key parameters were derived using established composite models. Table 1 summarizes the input parameters and governing relationships used for analysis, including the Rule of Mixtures for stiffness estimation and density-based expressions for volume fraction calculation. Table 2 presents selected mechanical and fracture-related parameters derived from experimental three-point bending data, including flexural modulus, estimated fiber stress, interfacial shear stress, and energy-related measures.

The parameters reported in Table 2 are not intrinsic material constants, but model-dependent estimates derived from classical composite mechanics approaches, including the Rule of Mixtures, shear-lag theory, and simplified fracture mechanics. These values are therefore contingent on assumptions related to stress distribution, interfacial behaviour, and damage evolution under bending, and are used primarily to support mechanistic interpretation. The experimentally obtained flexural modulus (18.64 GPa) represents an apparent bending stiffness derived from load–deflection response and may be influenced by specimen geometry, span-to-thickness ratio, and local stiffness effects. As such, it is not directly comparable to intrinsic elastic modulus predicted by micromechanical models. Similarly, the estimated interfacial shear stress and critical fibre length are subject to model assumptions and should be interpreted cautiously as indicative rather than absolute values. Accordingly, all model-derived parameters are distinguished from experimentally measured properties and are presented to qualitatively explain load transfer, interfacial performance, and observed failure mechanisms.

**Table 2:** Model-derived mechanical and interfacial parameters of silk–epoxy composites, including flexural modulus, estimated interfacial shear stress, critical fiber length, energy release rate, and total energy dissipation, obtained from experimental three-point bending data using classical composite theory models^5,10,17^.

Property	Symbol	Typical Unit	Value	Type
Flexural Strength	σ_f_	MPa	124 ± 5.7	Experimental
Strain at failure	ε_f_	-	0.025–0.040	Experimental
Flexural Modulus	E_flex_	GPa	5–6	Experimental
Fiber volume fraction	Vf	-	0.28	Calculated
Composite density	ρc	g/cm^3^	1.23	Measured
Theoretical Modulus (ROM)	Ec	GPa	5.55	Model (ROM)
Interfacial shear stress	τ_interfacial_	MPa	10–15	Model estimate
Critical Fiber Length (Apparent)	lc	µm	∼50–150	Model Estimate
Energy absorption (area under curve)	U	J	Qualitative/comparative	derived

### Three-point bending

**Fig. 3:**
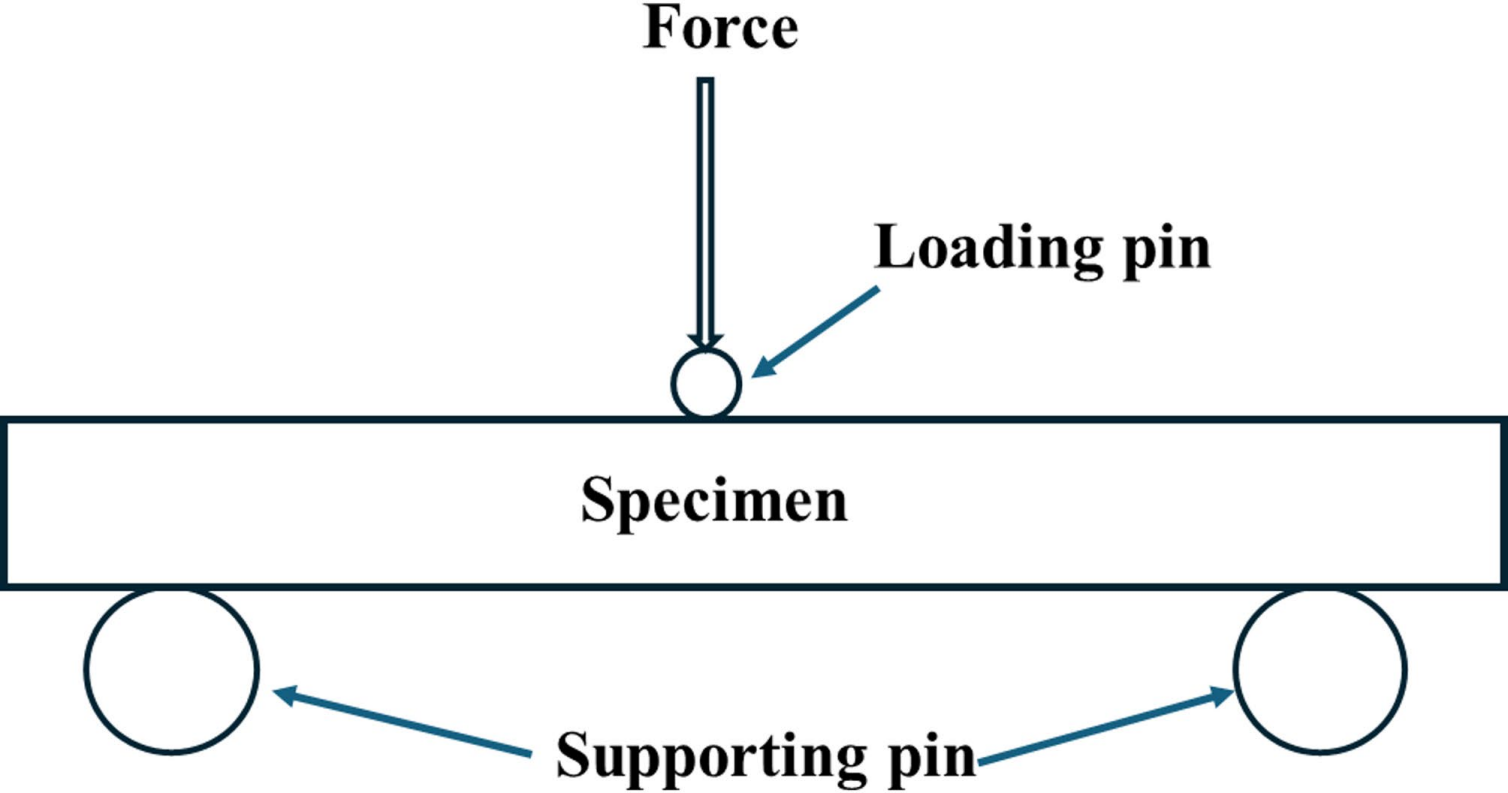
Schematic of the three-point bending test setup used to evaluate the flexural performance of silk fabric–reinforced epoxy composites in accordance with ASTM D790, showing specimen geometry, support span (60 mm), and central loading configuration for determining flexural strength and modulus.

Flexural testing was performed using an MTM-UTM Series Exceed E-43 universal testing machine with a 50 kN load capacity (Refer Fig. 3). The tests were conducted in accordance with ASTM D790 using a support span of 60 mm and a crosshead speed of 2 mm/min. The selected crosshead speed ensured quasi-static loading conditions, enabling stable stress transfer between the silk fibers and epoxy matrix while minimizing strain-rate effects. Three-point bending was selected to establish a well-defined stress state, with maximum tensile and compressive stresses occurring at the outer surfaces and a shear-dominated region at mid-thickness. This configuration facilitates identification of crack initiation beneath the loading nose, followed by propagation influenced by interfacial debonding, matrix cracking, and fibre bridging. Compared to four-point bending, this approach allows clearer correlation between localized stress distribution and microstructural damage mechanisms in heterogeneous natural fibre composites. Load and displacement were recorded continuously during testing. Flexural strength and modulus were calculated using standard ASTM relations. Strain was determined from mid-span deflection using beam theory, assuming small deflection conditions. The testing machine was calibrated prior to experimentation to ensure measurement accuracy. Three specimens were fabricated, of which two yielded complete and consistent load–displacement data suitable for analysis. Mechanical properties are reported as mean values with standard deviation, and the coefficient of variation is included to indicate variability. Given the limited sample size, the results are interpreted as indicative trends rather than statistically significant outcomes.

### Optical microscope

Fractographic examination of the silk–epoxy composite specimens was conducted using an Olympus BX53M optical microscope. Both bright-field and dark-field imaging modes were employed to characterize the microstructure and fracture features. Bright-field microscopy was used to observe overall morphology, including fiber distribution, resin-rich regions, and surface defects, while dark-field imaging enhanced contrast in regions with low optical density, enabling clearer identification of voids, interfacial discontinuities, and microcracks. This combined approach allowed qualitative and semi-quantitative assessment of microstructural heterogeneity, particularly variations in resin impregnation and void content. Image analysis was performed to estimate void fraction, which was subsequently correlated with mechanical performance. Void fraction was qualitatively assessed using optical image analysis, and comparative differences in void distribution were used to interpret variations in mechanical behavior, however, absolute void percentages were not quantified in the present study. All micrographs were acquired at specified magnifications with calibrated scale bars to ensure consistency and reproducibility of observations. Optical microscopy provides an effective intermediate-scale characterization technique, bridging macroscopic observations and higher-resolution SEM analysis, and is used here to support interpretation of damage initiation and propagation in relation to processing-induced features.

### Scanning electron microscope

Scanning electron microscopy was used to examine the fracture surfaces of the silk–epoxy composites after three-point bending. Prior to imaging, the specimens were sputter-coated with a thin gold layer to improve surface conductivity and minimize charging effects. Micrographs were obtained using a Zeiss EVO MA 18 microscope at multiple magnifications, with calibrated scale bars included in all images. The SEM analysis was used to identify and interpret dominant fracture mechanisms, including matrix cracking, interfacial debonding, fibre pull-out, and localized fibre fracture. Attention was given to features such as fibre–matrix interface quality, pull-out length, fractured fibre ends, and voids. These observations were analysed comparatively across specimens to assess the influence of microstructural variations on damage evolution. Where applicable, semi-quantitative assessment of fracture features was carried out, including relative extent of fibre pull-out and interfacial damage. The interpretation of these features was linked to load transfer behaviour under bending, considering tensile, compressive, and shear-dominated regions. The SEM results are therefore used not only for qualitative description but to support a mechanistic understanding of failure modes and interfacial performance.

## Results and discussions

### Stress versus strain diagram

**Fig. 4:**
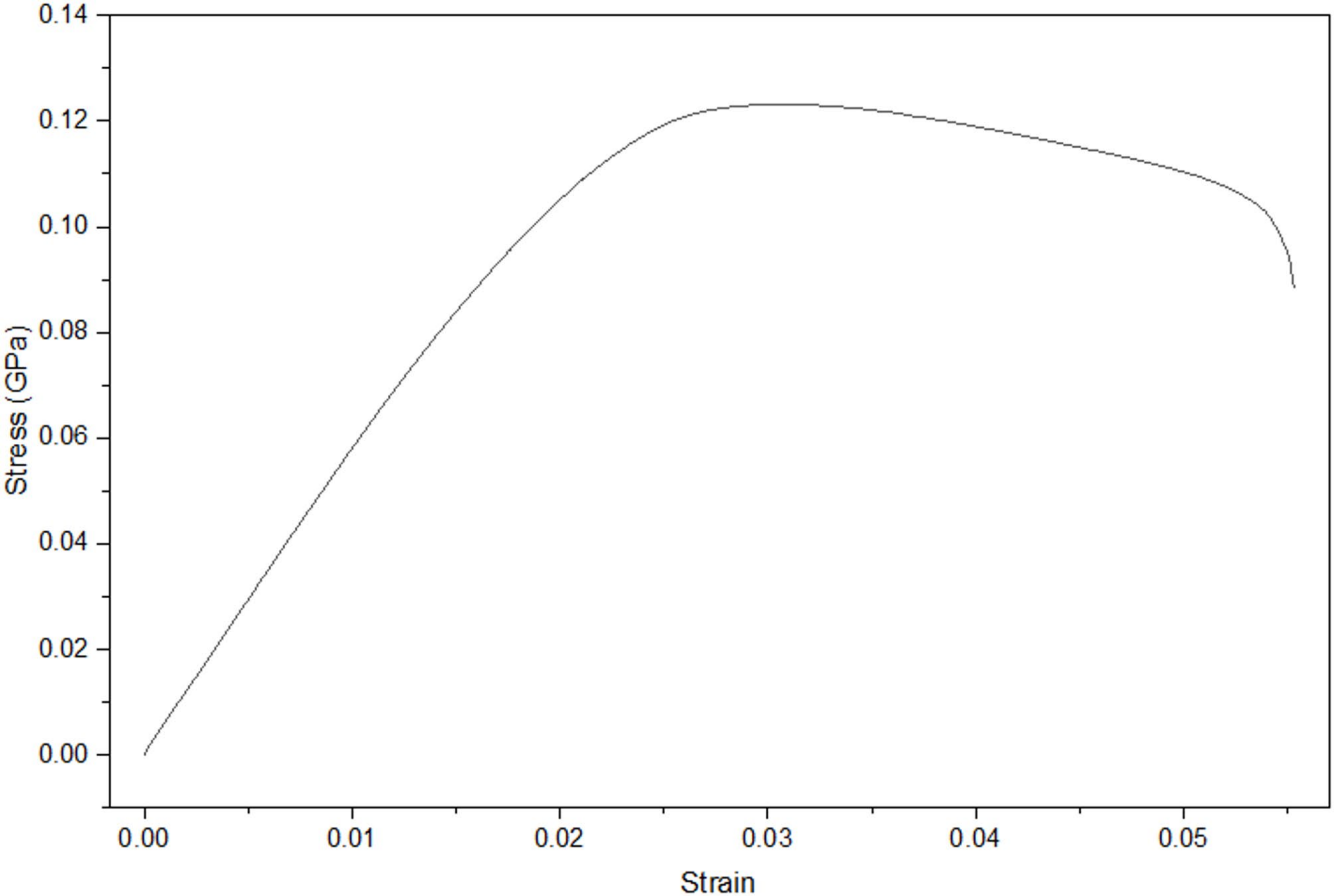
Three-point bending stress–strain response of silk–epoxy composite Sample 1 (specimen dimensions: 100 mm × 20 mm × 5 mm; support span: 60 mm), showing the initial elastic region, peak flexural stress (~ 120–130 MPa), and subsequent progressive post-peak softening under flexural loading.

The three-point bending stress–strain responses of the silk–epoxy composite specimens (100 mm × 20 mm × 5 mm; span = 60 mm) exhibit a nonlinear flexural behaviour typical of natural fiber–reinforced polymer systems. All samples show an initial near-linear elastic region, indicating effective stress transfer between the silk fibres and epoxy matrix. This region corresponds to elastic deformation where tensile stresses are highest at the outer surface and compressive stresses develop on the opposite face, with a neutral axis at mid-thickness (Refer Fig. 4).

With increasing strain, the response deviates from linearity, indicating the onset of damage mechanisms. This transition is associated with matrix microcracking, interfacial debonding, and shear stress concentration near the mid-thickness region. These mechanisms reflect the progressive breakdown of load transfer efficiency, which can be interpreted using a shear-lag framework where interfacial shear stress governs fibre engagement under bending.

**Fig. 5:**
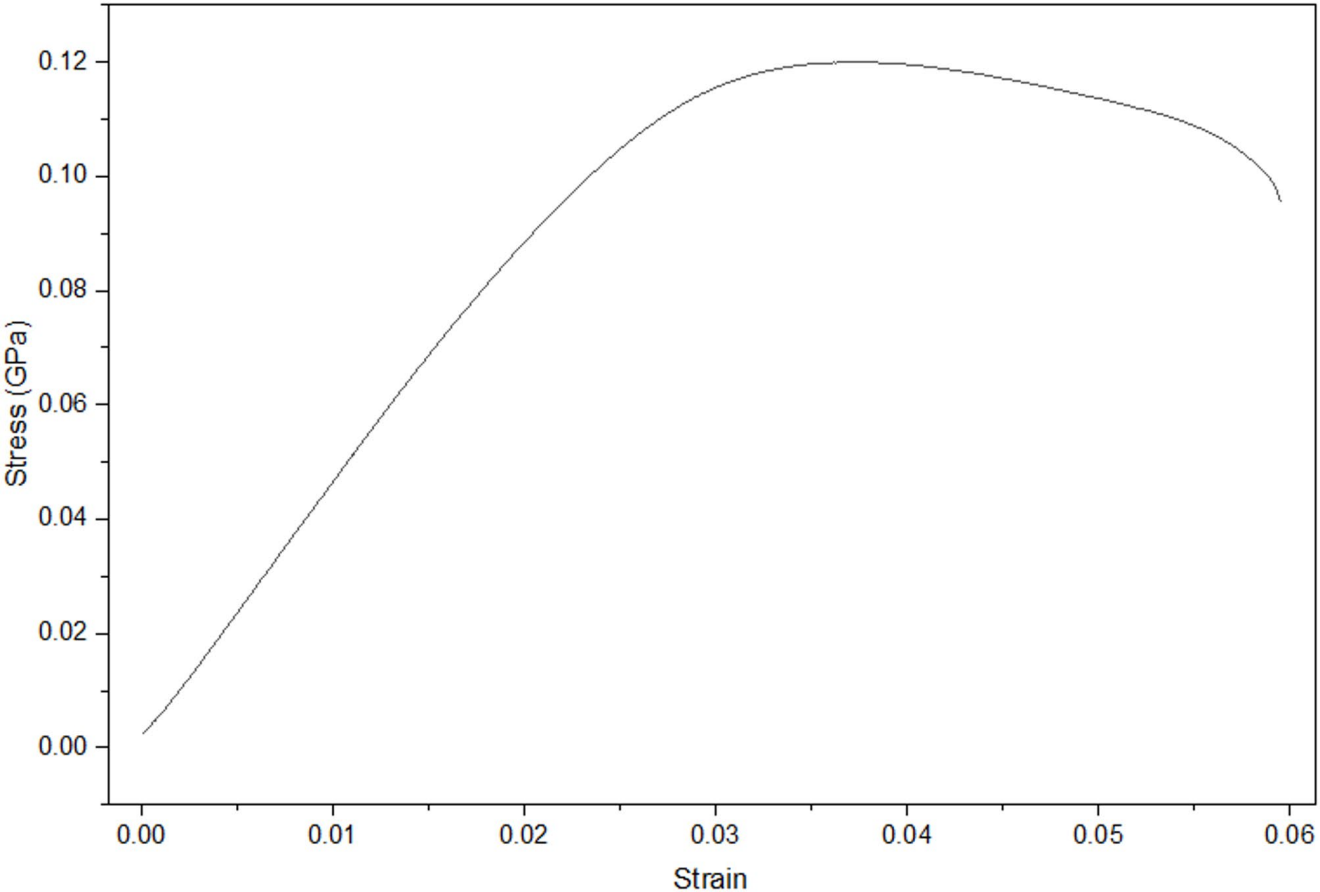
Three-point bending stress–strain response of silk–epoxy composite Sample 2 (specimen dimensions: 100 mm × 20 mm × 5 mm; support span: 60 mm), showing delayed peak strain and a more gradual post-peak softening behavior, indicating improved deformation tolerance under flexural loading.

**Fig. 6:**
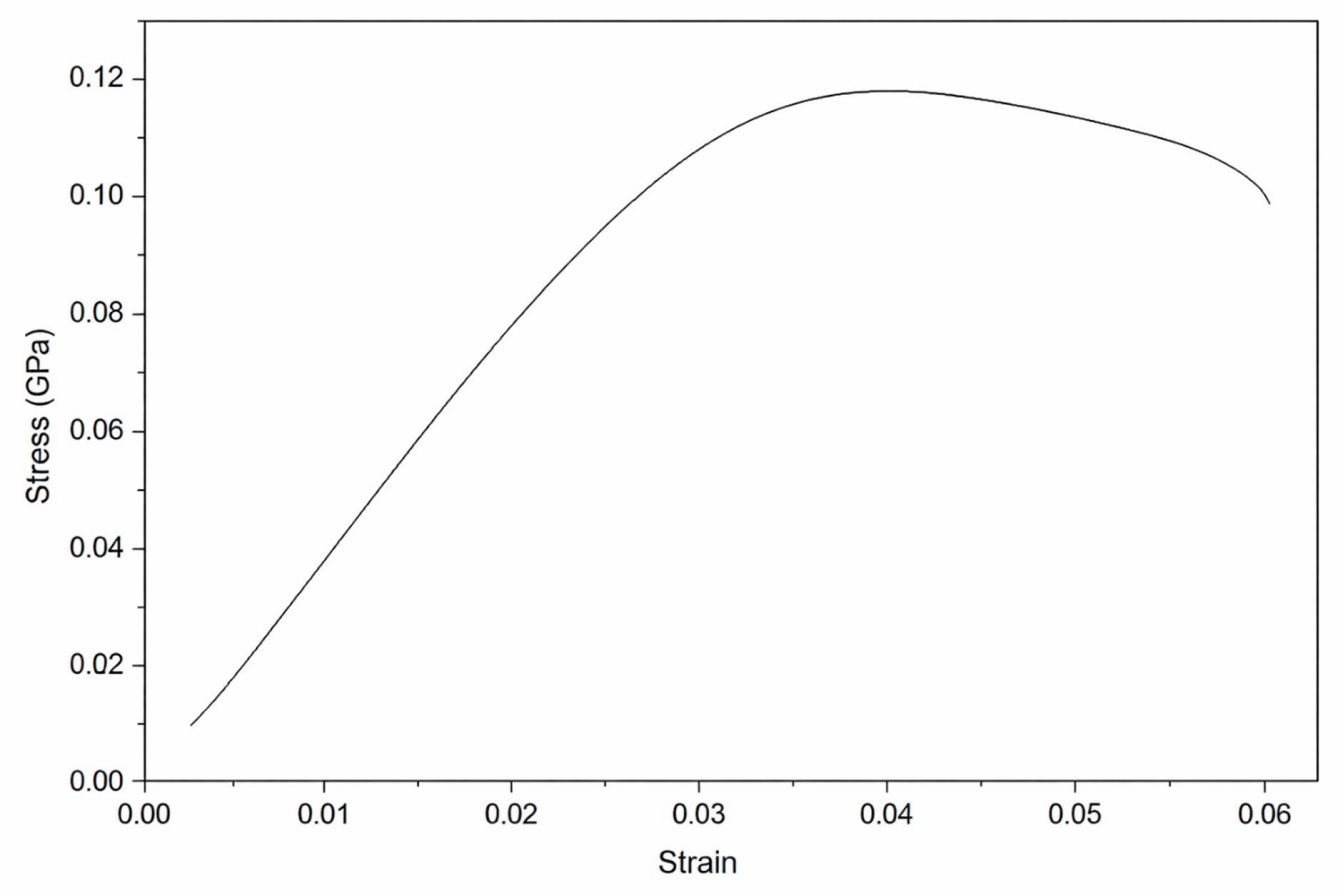
Three-point bending stress–strain response of silk–epoxy composite Sample 3 (specimen dimensions: 100 mm × 20 mm × 5 mm; support span: 60 mm), showing the initial elastic region, peak flexural stress (~ 120–130 MPa), and a controlled post-peak softening behavior indicative of progressive damage under bending.

All specimens reached peak flexural stresses in the range of 120–130 MPa at strains between approximately 0.025 and 0.040. The measured flexural strength (124 ± 5.7 MPa), obtained from the load–displacement response using ASTM D790 relations, is comparable to values reported for silk/epoxy systems (typically in the range of 100–150 MPa) [5,10], indicating that the present composites achieve similar load-bearing performance at moderate fiber volume fractions. Beyond the peak, a gradual reduction in stress was observed rather than abrupt failure, indicating a pseudo-ductile response. This post-peak softening is attributed to progressive damage mechanisms including matrix cracking, interfacial debonding, fibre pull-out, and localized fibre fracture. The sustained load-bearing capacity beyond peak stress suggests effective energy dissipation through fibre bridging and crack deflection (Refer Figs. 5 and 6).

Despite similar peak strengths, the three samples exhibited distinct deformation behaviour. Sample 1 showed earlier post-peak softening, suggesting localized defects such as voids or weak interfacial regions that promoted early crack initiation. In contrast, Sample 2 exhibited a more gradual stress decline and higher strain-to-failure, indicating improved interfacial integrity and more uniform stress distribution. Sample 3 demonstrated intermediate behaviour, with controlled stress reduction but less stability compared to Sample 2. These differences highlight that deformation tolerance is governed primarily by microstructural heterogeneity rather than strength alone.

The fracture process can be understood in terms of stress distribution under bending. Crack initiation occurs in the tensile region beneath the loading point, followed by propagation influenced by interfacial debonding and shear stresses near the neutral axis. Specimens with better impregnation and lower void content exhibit delayed crack initiation and more stable crack growth, whereas defect-rich regions promote rapid damage accumulation.

**Fig. 7:**
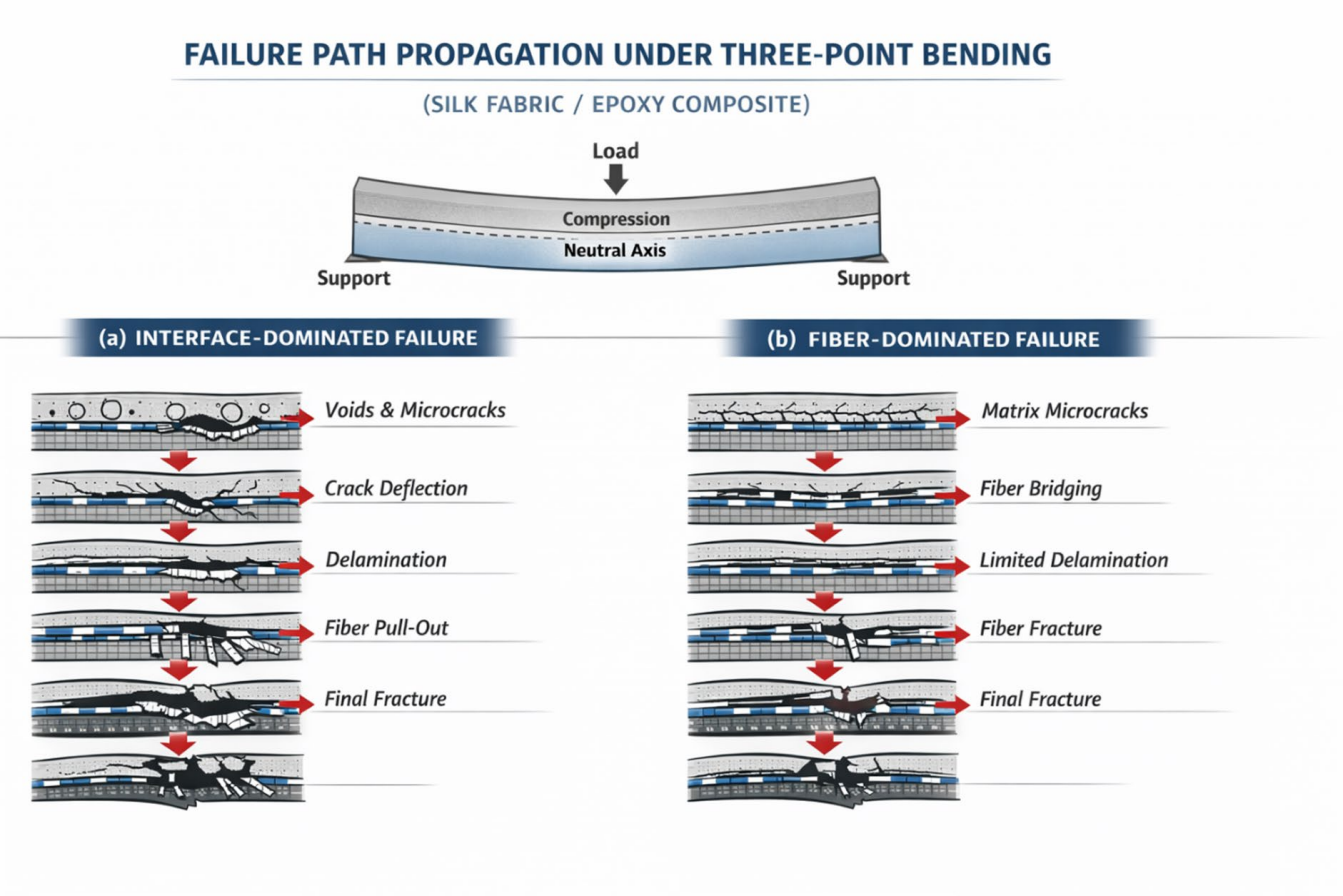
Schematic illustration of failure path propagation in silk–epoxy composites under three-point bending, showing crack initiation in the tensile region, propagation influenced by interfacial debonding and shear stresses, and the transition from interface-dominated damage (Sample 1) to fiber-dominated failure (Samples 2 and 3).

A schematic representation of failure pathways (Refer Fig. 7) illustrates this transition from interface-dominated to fibre-dominated failure. In Sample 1, weak interfaces and defects lead to early debonding and extensive fibre pull-out. In contrast, Samples 2 and 3 exhibit improved interfacial continuity, resulting in delayed crack initiation and more controlled failure progression. The stress–strain behavior demonstrates that silk–epoxy composites combine moderate flexural strength with progressive damage tolerance. The observed variations across samples emphasize the critical role of interfacial quality, void content, and processing-induced heterogeneity in governing flexural performance.

### Optical microscopy

**Fig. 8:**
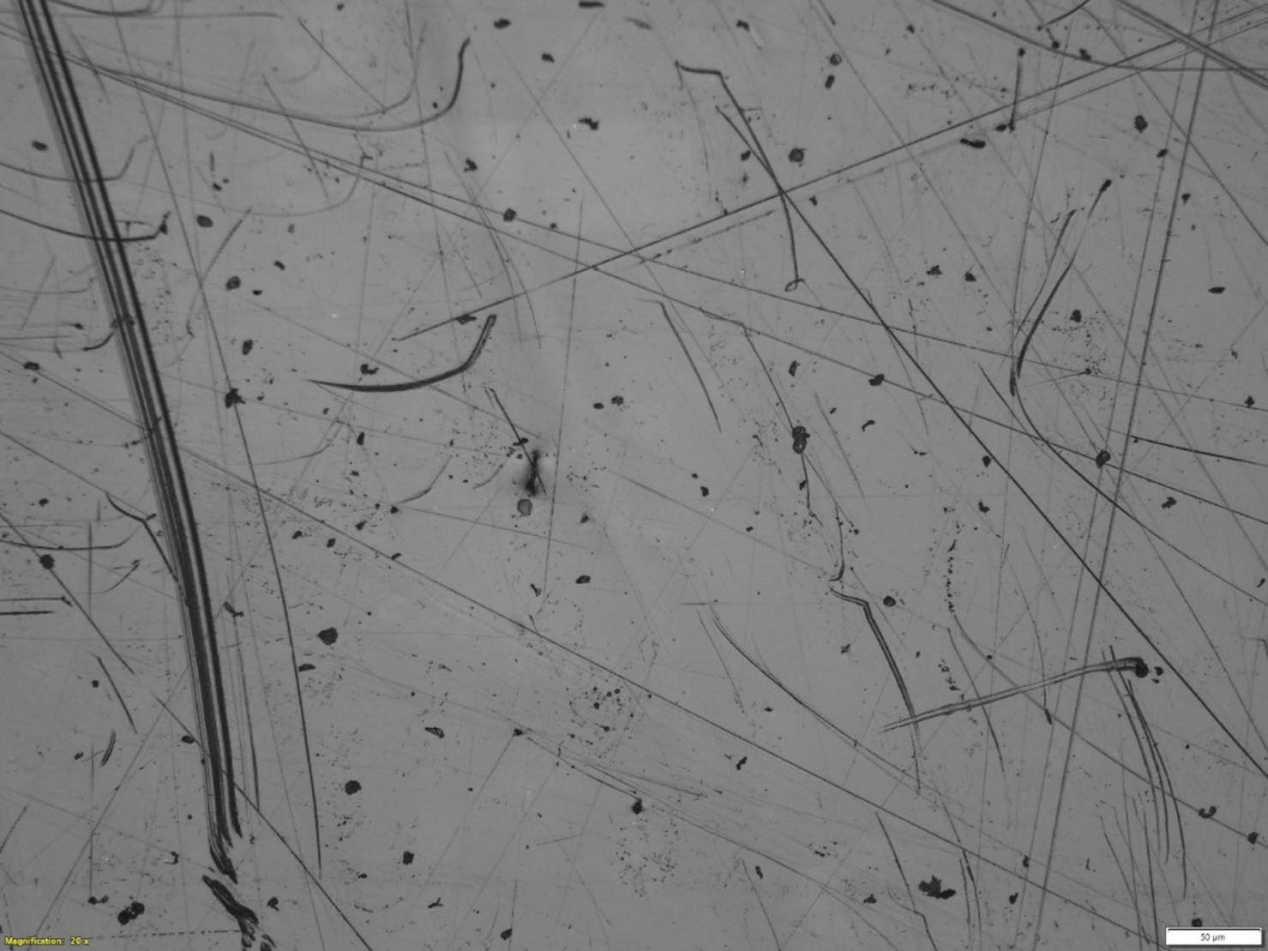
Optical micrograph (bright-field/dark-field) of the surface morphology of a silk–epoxy composite, showing fiber distribution, matrix-rich regions, microvoids, and localized fiber pull-out; features indicative of non-uniform impregnation and interfacial debonding (scale bar: 50 μm).

**Fig. 9:**
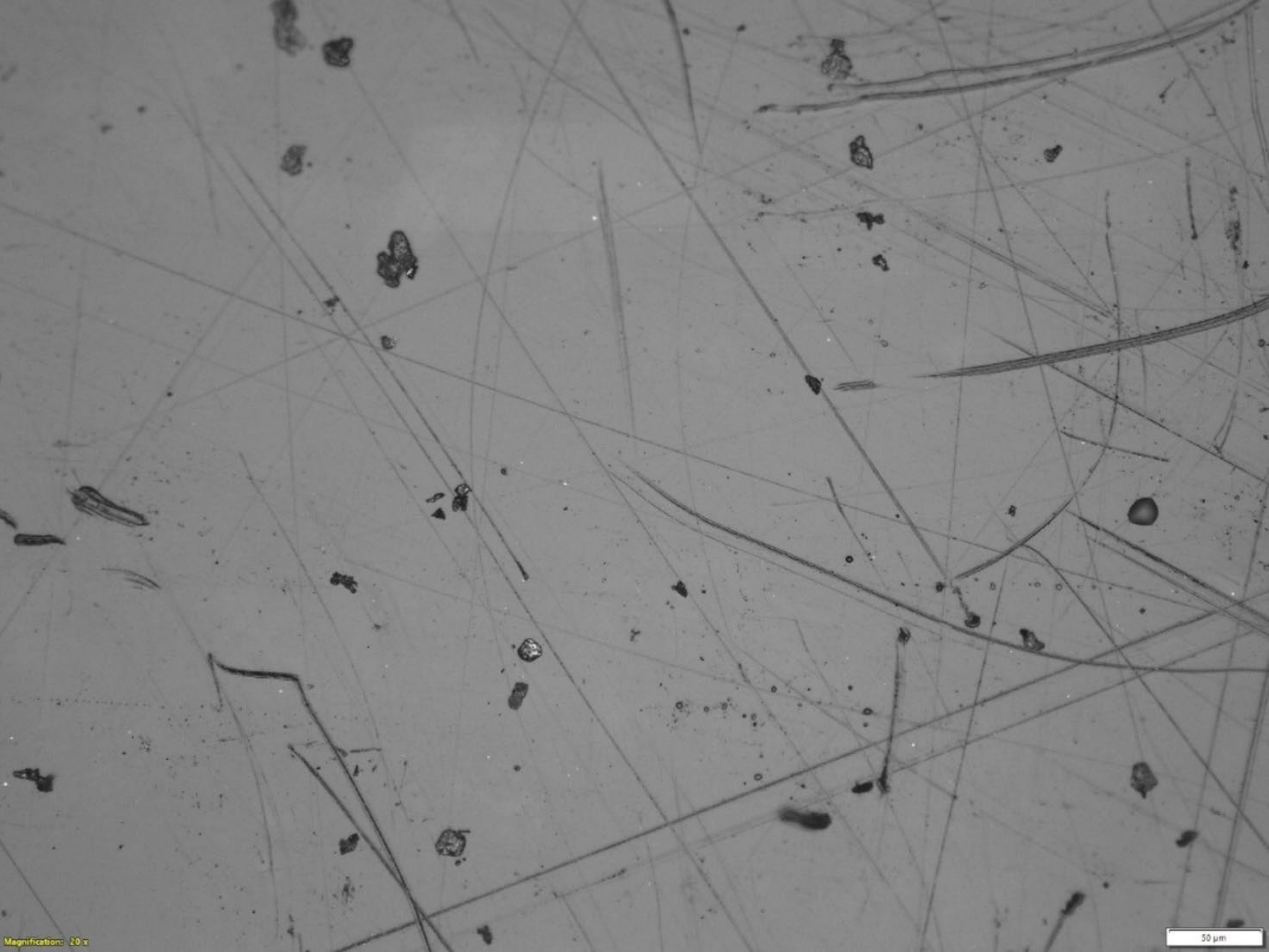
Optical micrograph (bright-field/dark-field) of the surface morphology of silk–epoxy composite showing relatively uniform fiber distribution, reduced fiber pull-out, and fewer interfacial voids compared with Sample 1, indicating improved resin impregnation and interfacial continuity (scale bar: 50 μm).

Optical microscopy was used to examine the surface morphology and fracture features of the silk–epoxy composites under both bright-field and dark-field imaging conditions. The combined use of these two modes enabled clearer identification of microstructural features, including fibre distribution, matrix continuity, voids, and damage evolution under flexural loading.

**Fig. 10:**
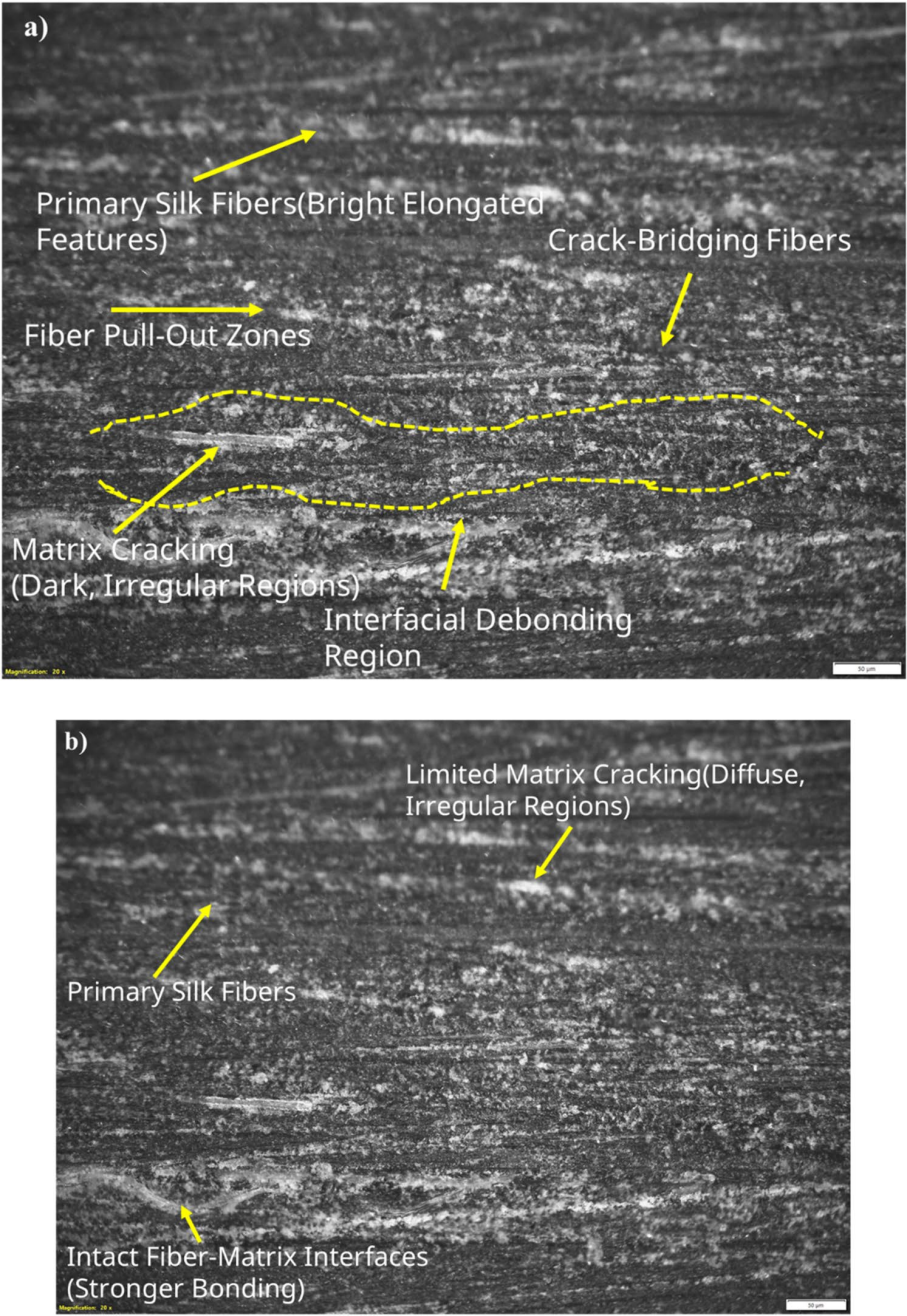
**(a)** Optical micrograph (dark-field) of silk–epoxy composite after three-point bending, showing matrix cracking, interfacial debonding, fiber pull-out, and a shear-dominated damage band near the mid-thickness, along with crack-bridging fibers that indicate progressive damage under flexural loading (scale bar: 50 μm). **(b).** Optical micrograph (dark-field) of silk–epoxy composite after three-point bending, showing limited matrix cracking, reduced fiber pull-out, and largely intact fiber–matrix interfaces, with crack-bridging fibers indicating improved interfacial integrity and more stable damage progression (scale bar: 50 μm).

The micrographs reveal a heterogeneous microstructure consisting of silk fibres embedded within the epoxy matrix, with variations in fibre distribution and resin impregnation across samples. In samples exhibiting lower processing quality (Refer Figs. 8 and 10(a)), the presence of micro-voids, resin-rich regions, and partially exposed fibres indicates non-uniform impregnation and possible air entrapment during fabrication [18]. These regions act as stress concentration sites, promoting early matrix cracking and interfacial debonding. Dark-field imaging highlights these defects more clearly, showing crack initiation along fiber–matrix interfaces and a distinct damage band near the mid-thickness, corresponding to the shear-dominated region in three-point bending. Extensive fibre pull-out and interfacial separation are evident, suggesting interface-dominated failure.

**Fig. 11:**
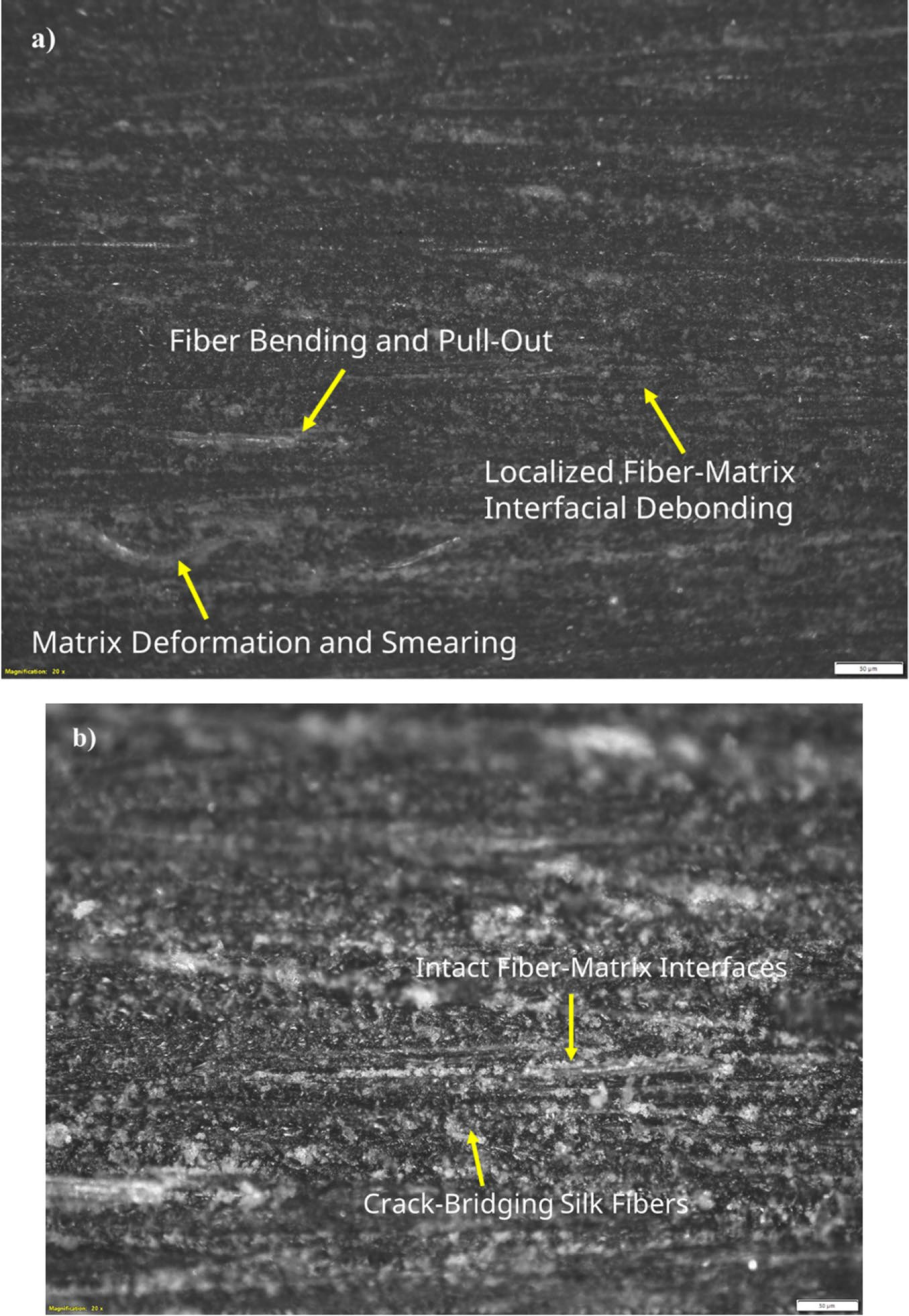
**(a)** Optical micrograph (bright-field) of silk–epoxy composite after three-point bending, showing matrix deformation and localized cracking, fiber bending and pull-out, and interfacial debonding consistent with progressive damage under flexural loading (scale bar: 50 μm). **(b).** Optical micrograph (bright-field) of silk–epoxy composite after three-point bending, showing limited matrix cracking, largely intact fiber–matrix interfaces, and crack-bridging fibers, indicating improved interfacial continuity and more stable damage evolution under flexural loading (scale bar: 50 μm).

In contrast, samples with improved processing conditions (Refer Figs. 9 and 10(b)) exhibit more uniform fibre distribution, reduced void content, and better matrix continuity. Both bright-field and dark-field observations indicate stronger fibre–matrix interfaces, with fewer instances of fibre exposure and interfacial gaps. Damage in these samples is more diffuse, with delayed crack initiation and more stable propagation. The presence of intact fibres bridging across cracks is clearly observed, indicating effective load redistribution and enhanced energy dissipation. Further observations under bright-field conditions (Refer Fig. 11(a) and Fig. 11(b)) confirm the progression of damage mechanisms. In defect-rich regions, matrix smearing, fibre misalignment, and interfacial gaps are prominent, indicating progressive debonding and localized deformation. In contrast, well-impregnated regions show limited cracking and intact interfaces, with clear evidence of crack bridging by silk fibres, which restricts crack growth and contributes to sustained load-bearing capacity [19,20].

The combined optical analysis demonstrates that void content, fibre distribution, and interfacial integrity govern damage initiation and propagation. Samples with higher defect density exhibit early crack initiation and unstable propagation, whereas improved microstructural uniformity leads to delayed damage and a more controlled, pseudo-ductile flexural response. These observations establish a direct link between processing-induced heterogeneity and the mechanical behaviour observed under three-point bending.

### Scanning electron microscopy

#### SEM images before three-point bending

**Fig. 12:**
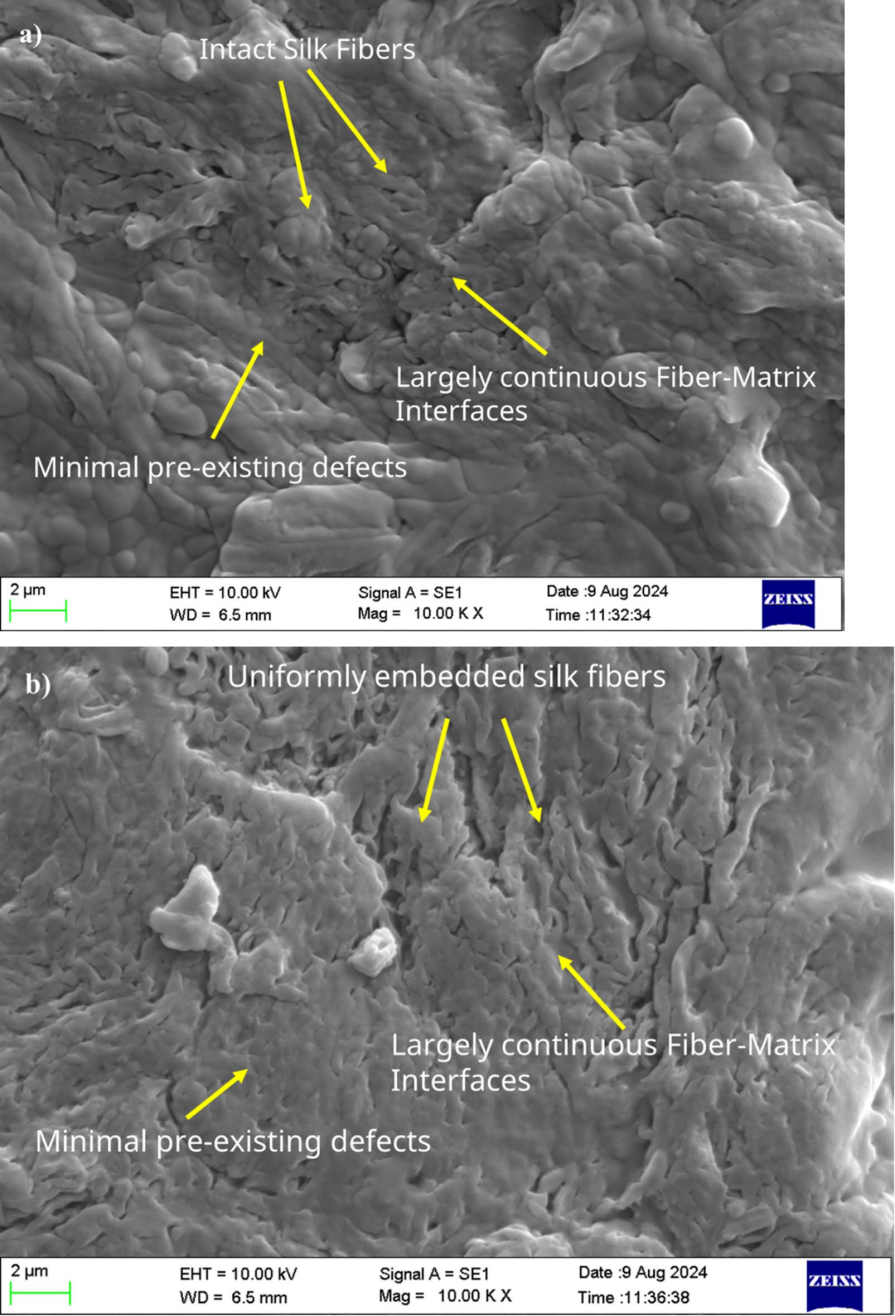
**(a)** SEM micrograph of silk–epoxy composite before three-point bending (Sample 1), showing intact fibers embedded within the epoxy matrix with largely continuous fiber–matrix interfaces and only minor surface irregularities, representing the undamaged pre-test microstructure. **(b).** SEM micrograph of silk–epoxy composite (Sample 2) before three-point bending, showing uniformly embedded fibers, a dense and continuous matrix, and minimal voids or interfacial discontinuities, indicating a well-consolidated and undamaged pre-test microstructure.

The SEM micrographs of the silk–epoxy composites prior to three-point bending show the initial, undeformed microstructure of the material (Refer Fig. 12(a) and Fig. 12(b)). In both cases, the silk fibres are embedded within the epoxy matrix with clearly visible contours, indicating effective impregnation during fabrication. The fibre surfaces appear largely smooth, and no load-induced damage such as cracking or deformation is observed, confirming that the specimens are in an undamaged state before mechanical testing. However, subtle differences in microstructural quality are evident between the samples. In Fig. 12(a), localized matrix irregularities and small void-like features are present, suggesting minor non-uniformity in resin flow and possible air entrapment during processing. These features may act as potential stress concentration sites under subsequent loading. In contrast, Fig. 12(b) shows a comparatively more uniform and dense matrix morphology, with fewer visible voids and improved fibre distribution.

The fibre–matrix interfaces in both samples appear largely continuous; however, the interfaces in Fig. 12(b) exhibit fewer discontinuities and better interfacial contact, indicating improved adhesion. This difference in interfacial integrity and defect distribution provides an important baseline for interpreting the mechanical response. Samples with lower defect density and more uniform interfaces are expected to exhibit delayed damage initiation and more stable stress transfer during bending, whereas localized defects may promote earlier crack initiation. The pre-bending SEM observations establish the initial microstructural condition of the composites and highlight the role of processing-induced heterogeneity. These differences are subsequently reflected in the flexural behaviour and fracture characteristics observed after three-point bending.

### SEM images after three-point bending

**Fig. 13:**
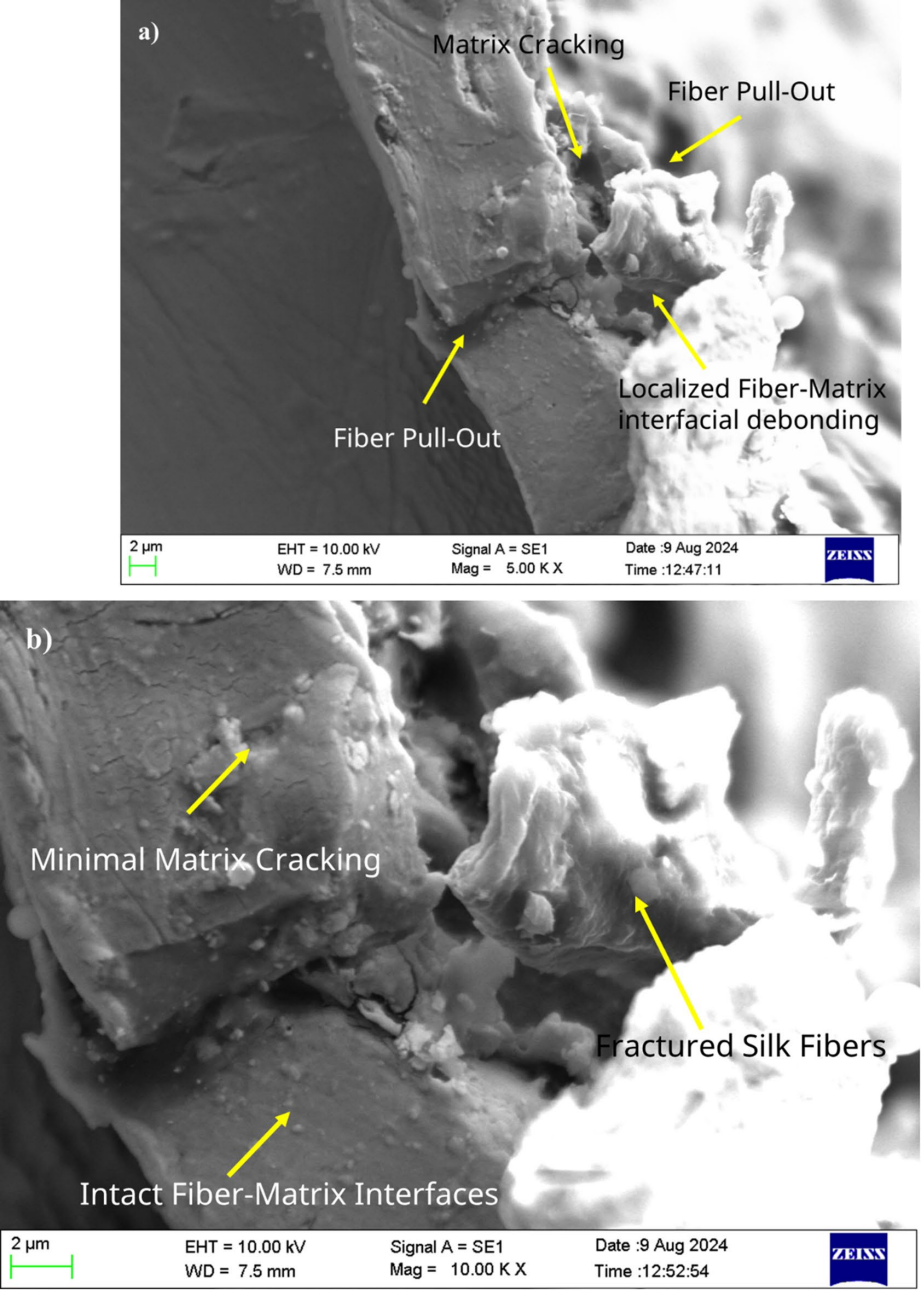
**(a)** SEM micrograph of silk–epoxy composite (Sample 1) after three-point bending, showing matrix cracking along fiber–matrix interfaces, fiber pull-out, and localized interfacial debonding, indicating interface-dominated damage and progressive failure under flexural loading. **(b).** SEM micrograph of silk–epoxy composite (Sample 2) after three-point bending, showing localized fiber fracture, limited matrix cracking, and largely intact fiber–matrix interfaces, indicating fiber-dominated failure and effective stress transfer under flexural loading (scale bar: 2 μm).

The SEM micrographs obtained after three-point bending reveal distinct fracture morphologies associated with flexural damage evolution (Refer Fig. 13(a) and Fig. 13(b)). In Fig. 13(a), extensive matrix cracking is observed, particularly along fibre–matrix interfaces, indicating that crack initiation occurred in regions subjected to high tensile and interfacial shear stresses. Several silk fibres exhibit pull-out and partial debonding, confirming that interfacial failure contributed significantly to damage progression. The presence of rough fracture surfaces, interfacial gaps, and coexisting fibre fracture suggests a mixed-mode failure involving matrix cracking, interfacial sliding, and localized fibre breakage. These features are consistent with the stress–strain response, which showed early deviation from linearity and gradual post-peak softening, reflecting progressive damage accumulation. In contrast, Fig. 13(b) shows a comparatively different fracture morphology characterized by localized fibre fracture with limited fibre pull-out. Matrix cracking is present but remains confined, without forming continuous crack paths. The fibre–matrix interfaces appear largely intact in surrounding regions, indicating more effective load transfer and delayed interfacial failure. This behaviour is consistent with improved strain tolerance and a more stable post-peak response observed during flexural testing.

**Table 3:** Proposed interfacial interaction mechanisms at the silk fibroin–epoxy interface, summarizing functional group interactions and their potential role in interfacial adhesion and stress transfer.

Interaction Type	Silk Fibroin Functional Groups	Epoxy Matrix Functional Groups	Interfacial Role
Hydrogen bonding	Amide (–CONH–), hydroxyl (–OH)	Ether (–C–O–C–), hydroxyl (–OH)	Enhance interfacial adhesion through reversible secondary bonding, improving stress transfer and crack resistance
Electrostatic/dipole interactions	Protonated amine (–NH₃⁺), polar peptide segments	Polar hydroxyl groups, epoxide ring dipoles	Promotes localized attraction at the fiber–matrix interface, stabilizing the interphase region
Potential localized covalent interaction	Nucleophilic amine (–NH₂), hydroxyl (–OH)	Epoxide rings (oxirane)	Potential interaction pathways (not experimentally verified) at the silk-epoxy interface, contributing to improved interfacial continuity under curing conditions

A comparative assessment of the two microstructures highlights a transition from interface-dominated to fibre-dominated failure. The specimen in Fig. 13(a) exhibits extensive interfacial debonding, fibre pull-out, and matrix cracking, suggesting reduced interfacial integrity and the presence of processing-induced defects such as voids or incomplete impregnation. In contrast, Fig. 13(b) demonstrates more uniform stress transfer, with failure governed primarily by fibre fracture, indicating improved interfacial continuity and reduced defect density. From an interfacial perspective, these differences can be interpreted in terms of the degree of physical and chemical compatibility between silk fibroin and the epoxy matrix. Silk fibroin contains polar functional groups (amide and hydroxyl), which can participate in secondary interactions such as hydrogen bonding with epoxy-derived groups. These interactions are considered plausible contributors to interfacial adhesion and stress transfer; however, they are not directly verified in this study and are discussed only as supporting mechanisms for interpretation.

While direct chemical bonding has not been experimentally confirmed, the interaction mechanisms summarized in Table 3 are consistent with established epoxy–amine systems and provide a conceptual framework for interpreting the observed interfacial integrity and pseudo-ductile flexural response Table 4.

**Table 4:** Comparative summary of microstructural observations from optical and SEM analyses.

Feature	Sample 1	Sample 2	Sample 3	Observation Method
Fiber distribution	Non-uniform	Relatively uniform	Moderately uniform	Optical
Void content	Higher	Lower	Moderate	Optical
Resin Impregnation	Incomplete	Improved	Moderate	Optical
Interfacial integrity	Weak	Strong	Moderate	SEM
Matrix cracking	Extensive	Limited	Moderate	SEM
Fiber pull-out	Significant	Reduced	Moderate	SEM
Fiber fracture	Limited	Localized	Moderate	SEM
Dominant failure mode	Interface-dominated	Fiber-dominated	Mixed	Combined

## Conclusions

This study investigated the flexural behaviour and microstructure–property relationships of silk fabric–reinforced epoxy composites fabricated under controlled processing conditions. The composites exhibited a pseudo-ductile flexural response, characterized by an initial elastic regime followed by progressive damage and gradual post-peak softening. The measured flexural strength ranged from 100 to 130 MPa (mean: 124 ± 5.7 MPa). The composites achieved a flexural strength of 100–130 MPa with observable pseudo-ductile behavior and progressive failure. Variations in strain-to-failure and post-peak stability across samples indicate that deformation behavior is governed primarily by microstructural integrity and interfacial quality rather than peak strength alone.

Multiscale characterization established direct correlations between processing-induced heterogeneity and mechanical response. Optical microscopy confirmed that void content, non-uniform resin impregnation, and fibre distribution influence crack initiation and propagation. SEM-based fractography identified dominant failure mechanisms, including matrix cracking, interfacial debonding, fibre pull-out, and localized fibre fracture. A transition from interface-dominated to fibre-dominated failure was observed in samples with improved interfacial continuity, resulting in delayed crack initiation and enhanced damage tolerance. These findings are consistent with bending stress distribution, where crack initiation occurs in the tensile region and propagation is influenced by interfacial shear stresses near the neutral axis.

The study contributes by (i) establishing a clear linkage between stress–strain behaviour and multiscale fracture features, (ii) demonstrating the role of moderate fibre reinforcement (approximately 20–40 vol%) in balancing processability and performance, and (iii) identifying processing-induced defects, particularly voids and interfacial discontinuities, as primary factors governing flexural stability. However, the findings are subject to limitations, including a small sample size, variability inherent to hand lay-up processing, and the absence of direct chemical or spectroscopic validation of interfacial interactions.

From an application perspective, silk–epoxy composites show potential for lightweight structures requiring moderate strength, energy absorption, and damage tolerance. At the same time, factors such as moisture sensitivity, environmental durability, and processing variability must be considered before practical implementation. Future work should focus on improving fabrication reproducibility, quantifying environmental effects, and employing spectroscopic techniques such as FTIR or XPS to validate interfacial mechanisms. Additionally, comparative evaluation under tensile and impact loading conditions would provide a more comprehensive understanding of mechanical performance.

## Data Availability

Data sets generated during the current study are available from the corresponding author on reasonable request.

## Declarations

### Supplementary Information

No supplementary information is available for this paper.
